# Free-Standing Selenium Impregnated Carbonized Leaf Cathodes for High-Performance Sodium-Selenium Batteries

**DOI:** 10.1186/s11671-019-2861-x

**Published:** 2019-01-18

**Authors:** Bingru Guo, Hongwei Mi, Peixin Zhang, Xiangzhong Ren, Yongliang Li

**Affiliations:** 1 College of Chemistry and Environmental Engineering, Shenzhen University, Shenzhen, 518060 Guangdong People’s Republic of China; 2 Guangdong Flexible Wearable Energy and Tools Engineering Technology Research Centre, Shenzhen University, Shenzhen, 518060 Guangdong People’s Republic of China

**Keywords:** Carbonized leaf, Free-standing, Binder-free, Sodium-selenium battery

## Abstract

A novel approach of carbonizing leaves by thermal pyrolysis with melt diffusion followed by selenium vapor deposition is developed to prepare the carbon-selenium composite cathodes for sodium-selenium batteries. The carbonized leaf possesses internal hierarchical porosity and high mass loading; therefore, the composite is applied as a binder- and current collector-free cathode, exhibiting an excellent rate capability and a high reversible specific capacity of 520 mA h g^−1^ at 100 mA g^−1^ after 120 cycles and 300 mA h g^−1^ even at 2 A g^−1^ after 500 cycles without any capacity loss. Moreover, the unique natural three-dimensional structure and moderate graphitization degree of leaf-based carbon facilitate Na^+^/e^−^ transport to activate selenium which can guarantee a high utilization of the selenium during discharge/charge process, demonstrating a promising strategy to fabricate advanced electrodes toward the sodium-selenium batteries.

## Introduction

With the rapid growth of electronic devices, sustainably rechargeable batteries are urgently needed, giving the urgent rise to exploit energy storage devices with high capacity and satisfactory rate performance [1–5]. Lithium ion batteries (LIBs) are the dominant power for electronic devices because of the advantages of high energy/power density and long-term stability [4, 6]. While the commercial LIBs cannot meet the future energy requirement of electric vehicles, lithium-sulfur (Li-S) batteries were greatly developed by the reasons of the low cost and high theoretical energy density of S [7–10]. However, the insulated nature of S and the dissolution of polysulfides are major challenges, leading to sluggish electrochemical reaction and low utilization of S, which hinders their practical applications [11–15].

Sodium ion batteries (SIBs) are considered to be a promising alternative for LIBs due to the low-cost and large-scale electrical energy storage applications [2, 16–19]. Especially, sodium-selenium (Na-Se) batteries have drawn increasing interest in these years [20–22]. The Se element is in the same group with S and has similar electrochemistry versus Na while the energy density of Na_2_Se (3254 mAh cm^−3^) is comparable to Li_2_Se (3467 mAh cm^−3^) [23–26]. Moreover, the electric conductivity of Se (10^−3^ S cm^−1^) is much higher than that of S (10^−30^ S cm^−1^ at 25 °C) [27]. The shuttle effect of polyselenides (which is similar to the polysulfides, Na_2_Se_*n*_, 3 < *n* < 8) can also deteriorate the cycle life of Na-Se batteries; therefore, it is a key challenge to overcome the hurdle of polyselenides shuttle [28–30]. Carbon matrixes with appropriate porosity and high electric conductivity, which are always used to load Se, have been regarded as an effective way to address the above issues in recent years [20, 21, 31, 32]. Much endeavor has been made to trap the soluble polyselenides within various carbons including carbon nanofibers [33, 34], carbon spheres [35, 36], and carbon nanosheets [22], which have been proved to effectively improve the electrochemical performance of Na-Se batteries. Nevertheless, the reported materials involve complex multistep processes and additional components (carbon black and binders); moreover, they are usually environmental harmful and economical costly.

Fortunately, renewable materials with remarkable properties provided by nature can meet our needs [5, 37]. For example, natural leaves are diversified with heteroatom-doping and exceptional porous structure and these natural hard carbons, which possess the impressive ability to store sodium ions, can act as alternative substitutes of traditional materials as electrode materials for SIB devices [32, 37]. The leaves of *Ficus* can be carbonized by thermal pyrolysis, and it is extremely satisfying that obtained leaves possess a hierarchical porous structure and moderate surface area. In brief, the porous voids can endow the pyrolysis products with high loading capacity and serve as ion-buffering reservoirs during electrochemical process, improving rate capability and power density [5, 38].

Herein, we prepared a new type of the free-standing Se impregnated electrode by melting diffusion followed Se vapor deposition into carbonized leaf which is obtained by thermal pyrolysis of natural leaves in a facile way. The highly reversible specific capacity (84% of the theoretical capacity of Se) is achieved for the first time when the biochar-selenium composite is applied as binder- and current collector-free cathodes for Na-Se batteries. In addition, the as-prepared composite electrode exhibits satisfactory rate capability and cycling stability. With the superiority properties, the carbonized leaf electrode demonstrated desirable electrochemical performance, which is a potential anode material for the Na-Se batteries.

## Methods

### Preparation of Carbonized Leaf

**Fig. 1:**
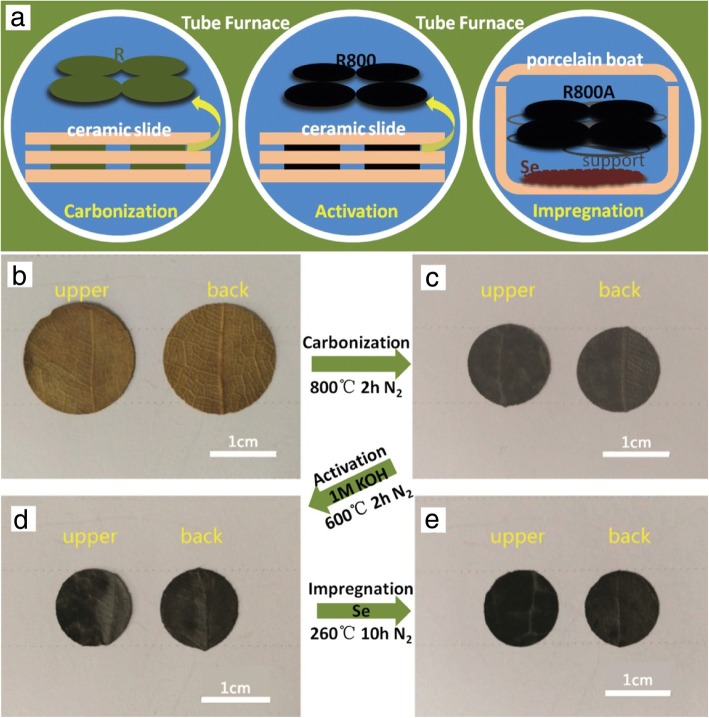
**a** Schematic illustration showing the preparation processes of the Se-R800A free-standing electrode. Digital photographs of **b** the dried R, **c** the R800, **d** the R800A, and **e** the Se-R800A

Dry leaves were cut into circular plates (17 mm in diameter). The leaf wafers were fastened between ceramic slides to avoid curling or pulverization during the carbonization process as shown in Fig. 1a. The leaf wafers were put into the tube furnace to carbonize at 800 °C for 2 h with a ramping rate of 5 °C min^−1^ under N_2_ flow. The carbonized leaf (denoted as R800) was immersed in 3.0 M HCl for 12 h to remove the inorganic salts. The R800 specimens were immersed in 1.0 M KOH for 12 h, then put into the tube furnace and activated at 600 °C for 2 h with a ramping rate of 5 °C min^−1^ under N_2_ flow to obtain porous materials (denoted as R800A). The samples were washed with deionized water several times and dried at 70 °C overnight in vacuum oven.

### Preparation of Se-R800A

Se powder was put on bottom of the porcelain boat, and the free-standing R800A films were suspended by an irony support in midair right above the Se powder, and the weight ratio of Se:C is not less than 2:1 in order to ensure excess Se powder as shown in Fig. 1a. Then the Se was melted at 260 °C under N_2_ atmosphere and maintained for 10 h to ensure a good penetration of Se. The weight of Se in the final Se-R800A electrode was measured by thermogravimetric analysis.

### Materials Characterization

The morphology and microstructure were observed by the scanning electron microscopy (SEM, Hitachi SU-70), the field emission scanning electron microscopy (FESEM, JSM-7800F, and TEAM Octane Plus), and transmission electron microscopy (TEM, JEM-2100, and X-Max80). The structure and Raman spectra were collected on the X-ray diffraction (XRD, PANalytical Empyrean with Cu-Kα radiation) and Raman microscope (Renishaw, inVia), respectively. Thermogravimetric analysis (TGA, STA409PC) was tested from room temperature to 700 °C with a heating rate of 10 °C min^−1^ under N_2_ atmosphere. BELSORP-max Surface Area and Porosimetry instrument was used to measure the N_2_ adsorption/desorption isotherms of electrodes. X-ray photoelectron spectroscopy (XPS) tests were carried out using a Thermo K-Alpha^+^ system.

### Electrochemical Measurements

The electrochemical tests were carried out using CR2032 coin cells, which were assembled with manual Na foil prepared by tableting press as the counter electrode inside an argon-filled glove box (MBRAUN, UNILab2000) with moisture and oxygen levels lower than 1 ppm. Glass fiber (Whatman) was used as the separator. The electrolyte was 1 M of NaClO_4_ in a mixture of ethylene carbonate /diethyl carbonate (EC/DEC, 1: 1 in volume). The free-standing Se-R800A was directly used as the working electrode without any binder and carbon conductor. The cyclic voltammogram (CV) measurements were performed on an electrochemical workstation (CHI660D). The galvanostatic charge-discharge tests were carried out over a voltage range of 0.005–3.0 V (vs. Na^+^/Na) on a battery test system (Land, CT-2001A). Electrochemical impedance spectroscopy (EIS) measurements were tested using the electrochemical workstation (CHI 760D) by applying a voltage of 5 mV over a frequency of 10^−2^–10^5^ Hz. The galvanostatic intermittent titration technique (GITT) test was performed by the discharge/charge process of the cells for 10 min at 10 mA g^−1^ and followed by a 40-min relaxation at most 50 cycles. All the cells were held at room temperature for at least 12 h before tests. All the specific capacity in this work was calculated on the basis of the loading Se weight. For the ex situ SEM tests, tested electrodes were carefully washed with DEC solvent for three times and dried overnight in vacuum oven.

## Results and Discussion

The Se-R800A free-standing electrode was fabricated by carbonization, KOH activation, and Se impregnation processes, which is presented in Fig. 1.

After carbonization process at 800 °C, the size of R800 (Fig. 1b) barely shrank (17 mm to 12 mm in diameter) and the thickness changed hugely (800 μm to 240 μm) with the weight loss of 74%. Figure 1c shows the R800 turned into black indicating that R was successfully transformed into carbon. After activation process, the weight of R800 continued to decrease ~ 9%. However, after Se impregnation process, the weight of R800A (Fig. 1d) increased 90% to transform into the Se-R800A as shown in Fig. 1e. It is noteworthy that the R800A films suspended in midair right above the Se powder were surrounded by Se vapor. This is an original idea of melt diffusion and vapor deposition due to avoiding isolated stray of Se in carbon matrixes [20]. Finally, the Se-R800A maintains well mechanical strength as a free-standing electrode for Na-Se batteries.

**Fig. 2:**
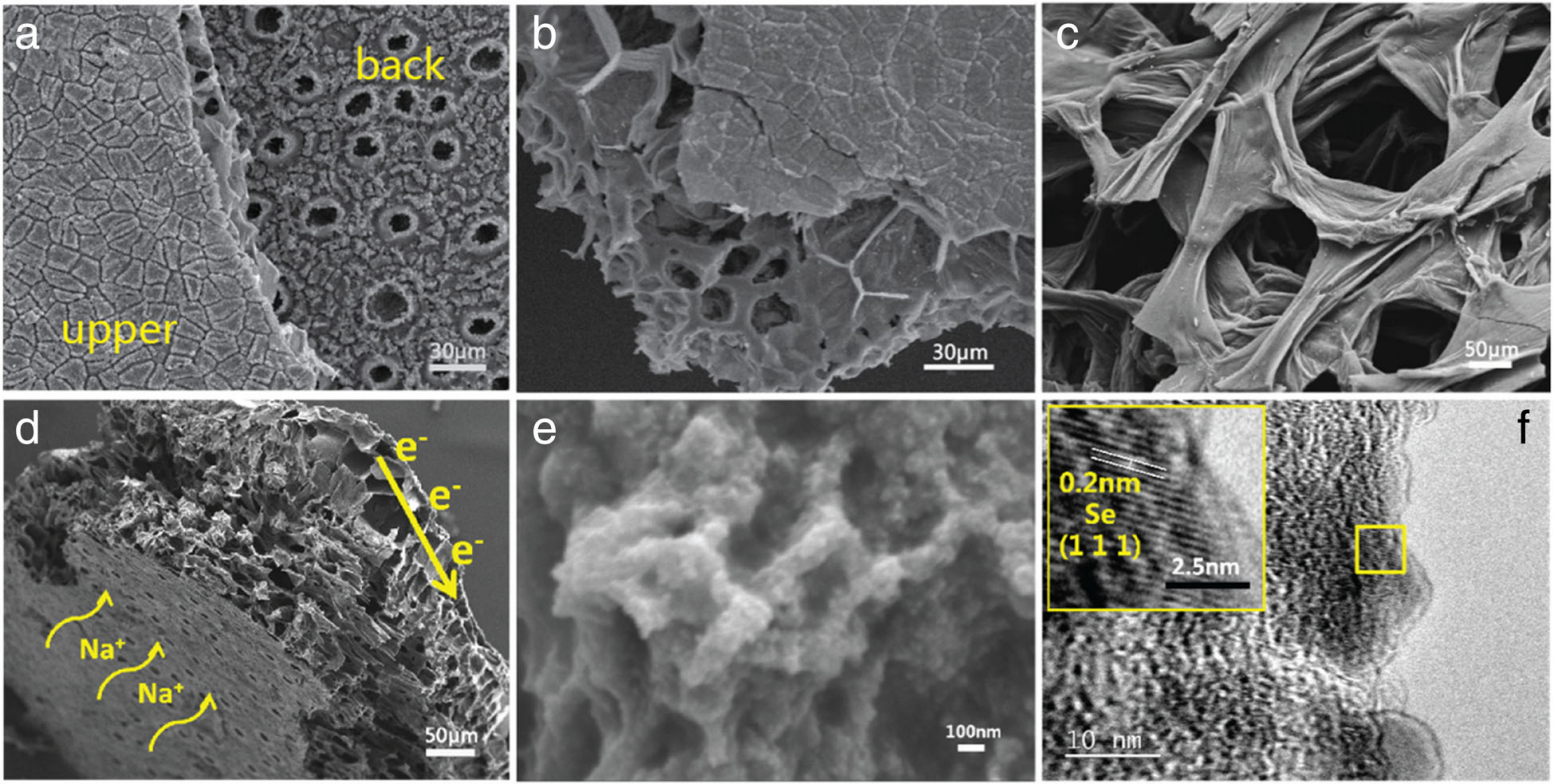
SEM images of the R800 **a** upper surface and back surface and **b** cross-sectional view. **c** Magnified SEM image of the carbon sheet in the sponge layer of the R800A. **d** FESEM image of the cross section. **e** Magnified FESEM image. **f** HRTEM image of the Se-R800A

Figure 2a shows the typical structure of a natural leaf with two different surfaces, where the upper surface is flat while the back surface contains uniform stomata. Figure 2b shows that the cross section of the leaf is plentiful porous with palisade and sponge cells inside to build enough space for the exchange of O_2_ and CO_2_ [37]. The carbonized leaf shows similar structure to the original porous structure of leaf; therefore, the whole structure with hierarchical porosity is suitable for the storage of sodium ions. Figure 2c shows the inside of the carbonized leaf where is filled with reticulated sheets overlapped. The thickness of the interconnected sheet is less than 100 nm, which can facilitate the electrolyte infiltration and shorten diffusion length for the ions. The whole thickness of the Se-R800A is about 240 μm as shown in Fig. 2d, and the stomata of the back surface provides enough channels for the electrolyte and Na^+^ ions to pass the arranged sponge layer of the carbonized leaf, then enter through the overlapping carbon sheets filled with Se (Fig. 2e) to fulfill the main electrochemical reaction and this layer concatenates with a well-aligned palisade layer. The upper surface is taken as the current collector and electrons travel along the conductive carbon sheet and then are collected by the upper surface layer [37]. Figure 2f shows further insight into the microstructure of the Se-R800A, where some Se particles and amorphous carbon were found. The inset image shows the lattice fringes for the ordered region measured by 0.2 nm, which could be attributed to the (111) crystal plane of Se. The Se-R800A with multilayered leaf structure aims at significantly mitigating the shuttle effect to improve long-term cycling and activating Se to ensure high Se utilization, which will improve the electrochemical performance of Na-Se batteries.

**Fig. 3:**
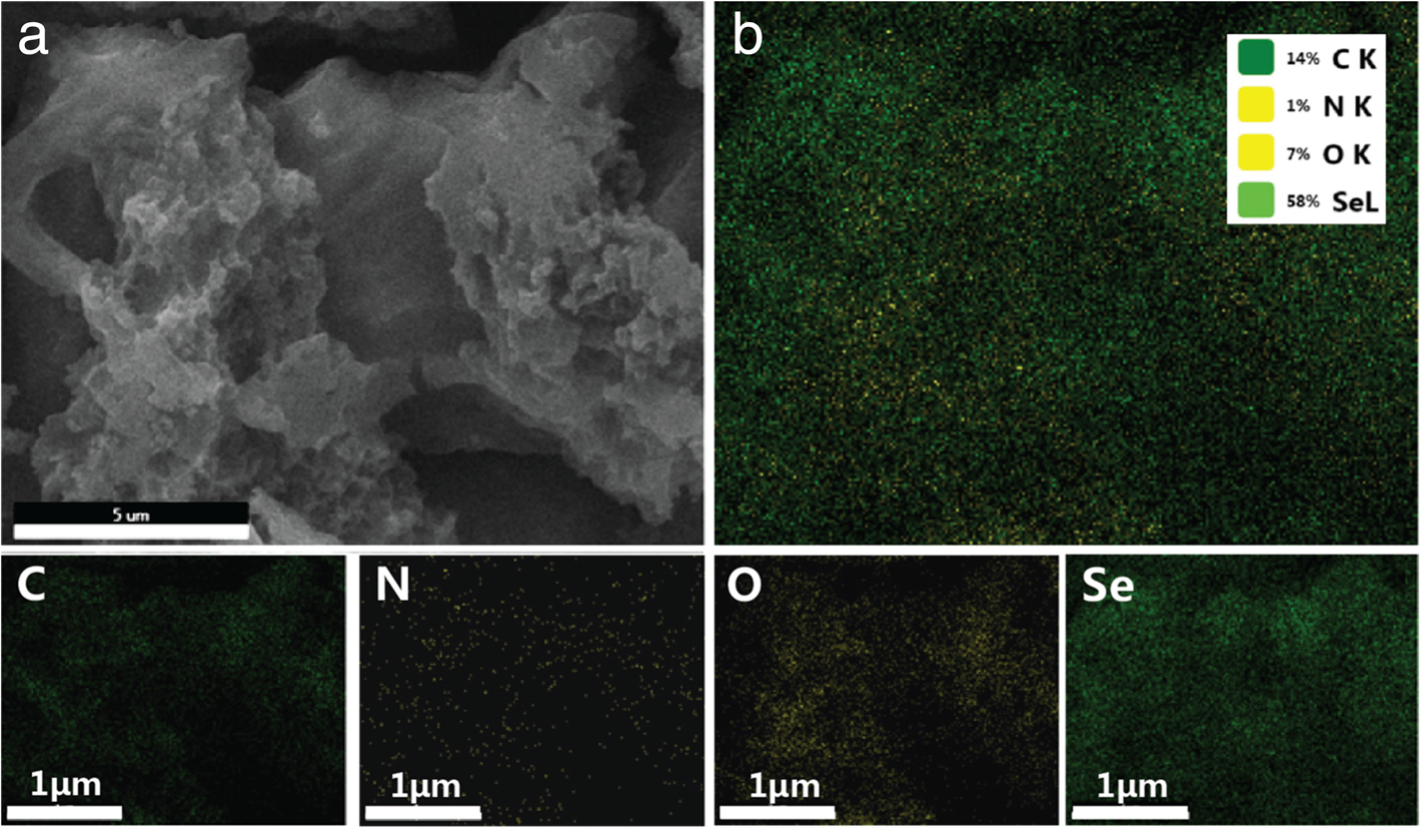
**a** FESEM images of the Se-R800A. **b** Elemental mapping images of C, N, O, and Se of the Se-R800A and the corresponding EDX results

As shown in Fig. 3a, the Se-R800A maintains the morphology of R800 and no isolated Se could be observed, while the EDX elemental mapping of the Se-R800A verifies the homogeneous distribution of Se as demonstrated in Fig. 3, which proves complete penetration of Se into the R800A. It confirms C-Se composites have been successfully manufactured. The Se signal is uniform through the cross section, the Se-R800A with a corresponding C, N, and O element mappings in the same region (Fig. 3b). As mentioned above, heteroatom-doping of N and O originated from biochar makes for facilitating electrochemical process and settling polyselenides [6, 39–41].

**Fig. 4:**
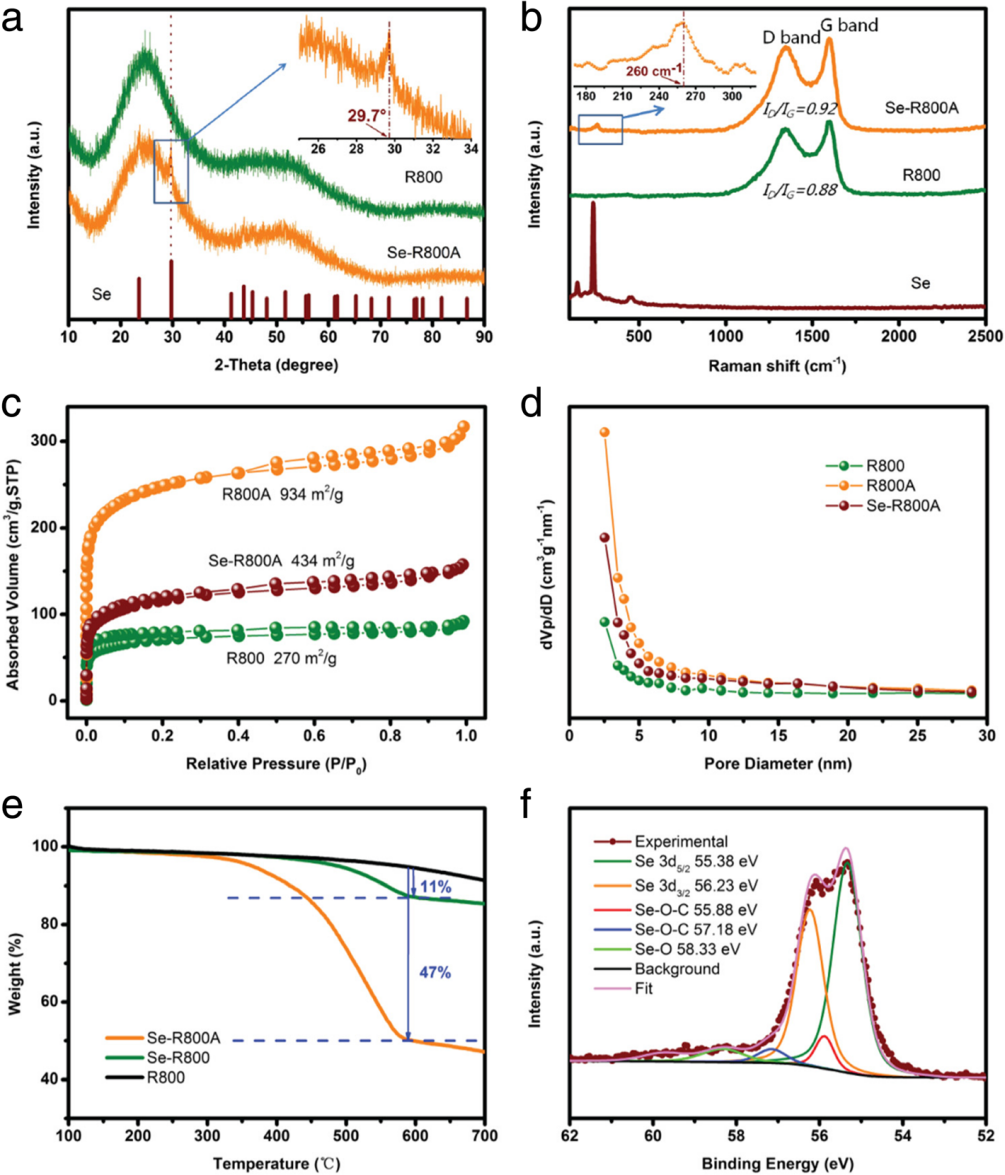
**a** XRD patterns and **b** Raman spectra. **c** N_2_ adsorption/desorption isotherms and **d** pore-size distribution curves obtained by the DFT method. **e** Thermogravimetric analysis. **f** XPS spectra of Se in the Se-R800A

To further study the structure of the Se-R800A, R800, and Se powder, the XRD patterns are shown in Fig. 4a. After Se infiltrating into R800A, the diffraction peaks of crystalline Se in Se-R800A mostly disappear and only amorphous humps (resemble the R800) could be seen, implying a full dispersion of amorphous Se into the R800A. The amorphous Se is proved to facilitate cycling stability and retard formation of soluble polyselenides in carbonate-based electrolyte [21, 22, 31]. However, the magnified peak at 29.7° (the inset in Fig. 4a) in Se-R800A is clearly observed, demonstrating a small quantity of crystalline Se still exists. Raman spectroscopy was applied to investigate the three samples. As indicated in Fig. 4b, the raw Se displays a sharp peak located at 234 cm^−1^, which is corresponding to the equilibrium trigonal Se [27]. However, for Se-R800A, these characteristic peaks disappear, and leaves a broad peak at 250–300 cm^−1^ on account of C-Se stretching vibration and the C-Se-Se group vibration [21]. The weak Se peak intensity is blue-shifted to 260 cm^−1^ (the inset in Fig. 4b), which is associated with the transformation from crystalline to molecular of Se [22, 24, 28, 39]. In addition, both R800 and Se-R800A feature the D-band at 1346 cm^−1^ and the G-band at 1598 cm^−1^, relating to the disordered and graphitic carbon, respectively. The intensity ratio of *I*_D_/*I*_G_ in Se-R800A is about 0.92 and higher than 0.88 of R800, revealing that Se insets and impacts the graphitization of the R800A but maintaining excellent electric conductivity [32]. This further confirms the Se-R800A can be used as a satisfactory cathode for Na-Se batteries. To examine the effects of KOH activation and Se impregnation, Fig. 4d demonstrates the surface structure of the R800, R800A, and Se-R800A, which all are the type IV isotherm similar to the adsorption of the microporous materials [31]. The Brunauer-Emmett-Teller (BET) calculated surface area is 270, 934, and 434 m^2^ g^− 1^ respectively, revealing that KOH is able to enlarge specific surface area by pore-creating to effectively trap Se [5]. Remarkably, after the Se impregnation, the specific surface area of the Se-R800A decreases by 54% in Fig. 4c, accompanied by a remarkable decrease in pore-size distribution in the range of 0.5–2 nm as shown in Fig. 4d, implying the diffusion of Se into the micropores of the R800A. These abundant micropores have been confirmed to effectively confine amorphous Se in the previous report [22, 27, 42]. The TGA is applied for the sake of affirming the loading weight of Se in the final composite material. The Se in Se-R800A began significantly evaporating at 300 °C, resulting in 47% Se weight loss by 550 °C. As illustrated in Fig. 4e, the Se-R800 presents the similar curve of the Se-R800A but only 11% Se loading is obtained, suggesting it is necessary to activate biochar and form micropores by KOH for loading Se. Up to 700 °C, R800 exhibited slight weight loss (< 2%) caused by the deep thermal pyrolysis and further graphitization. The chemical state of Se was further investigated by XPS as indicated in Fig. 4f. The 3d peak of Se is split into 3d_3/2_ and 3d_5/2_ with binding energies of 56.23 and 55.38 eV, respectively. These are slightly higher than those of crude Se 3d (55.95 and 55.15 eV), indicating the intense chemical interaction between Se and R800 matrix [34]. Generally, the R800 as biomass possesses heteroatoms (e.g., N and O as shown in Fig. 3 for N, O), especially O, offers strongly binding between Se and R800. This is proved by the appearance of Se-O (58.33 eV) peak in the spectrum. Obviously, two new peaks centered at 57.18 and 55.88 eV appear for the Se-R800A composite, indicating the generation of Se-O-C bonds during the impregnation process. The new peaks could be implied the formation of Se-O-C bonding, which leads to lower electron density of O site. This chemical bridging bond (-O-) endows C strongly to couple with Se and suppresses the shuttle effect of the polyselenides during cycling [24, 27, 39, 43].

In order to evaluate the electrochemical performance of the Na-Se batteries, the Se-R800A was directly used as the cathode in CR2032 coin cell. It is worthy to mention that the back surface of Se-R800A faces the metal Na and upper surface is as the current collector.

**Fig. 5:**
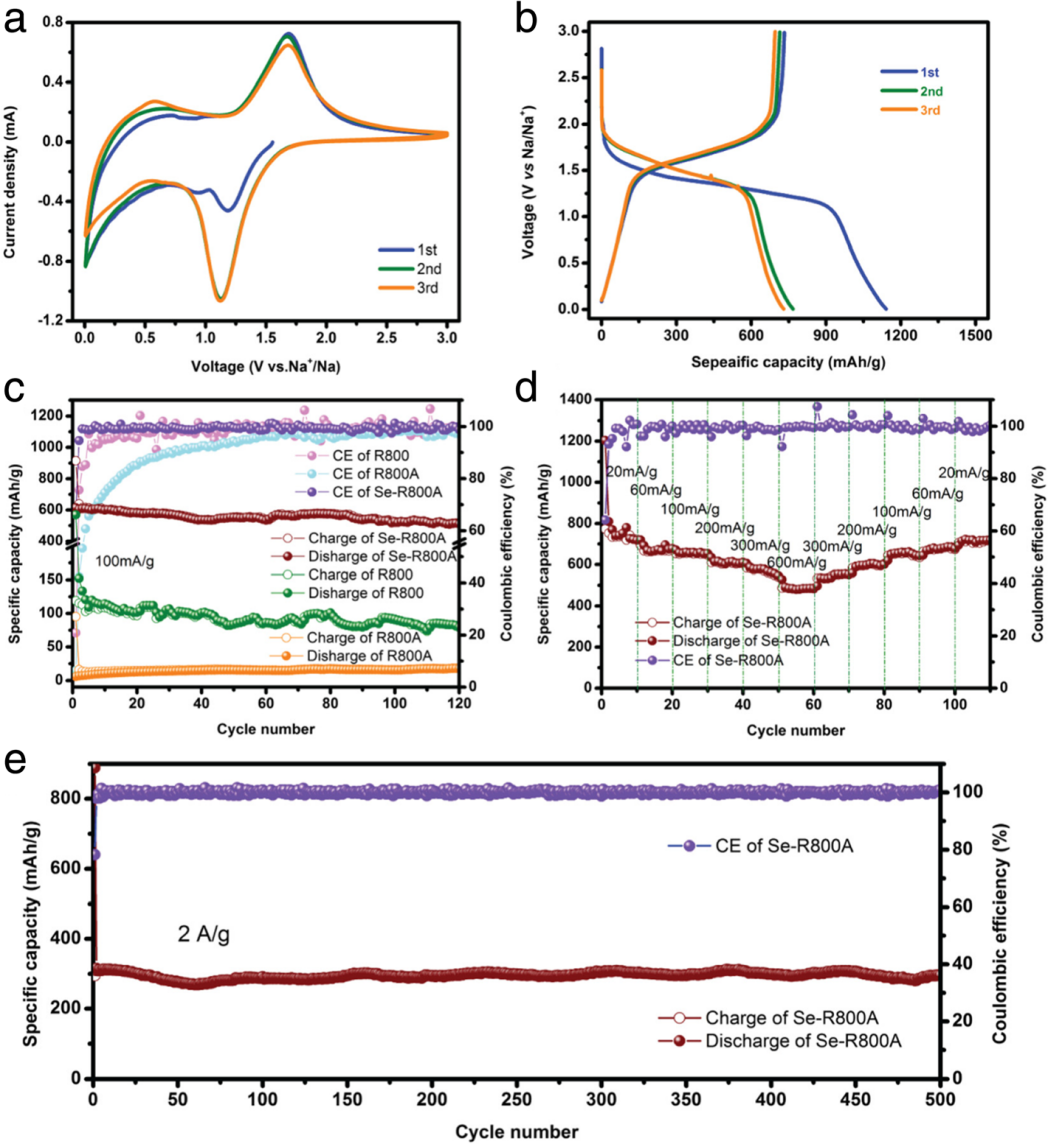
The electrochemical performance of the Se-R800A cathode in Na-Se batteries, **a** the CV curves at scan rate of 0.2 mV s^−1^, **b** the galvanostatic discharge/charge voltage profiles tested at 50 mA g^−1^, **c** the cycling performance of the Se-R800A, R800A, and R800 at 100 mA g^−1^, **d** the rate capability at various current densities, and **e** the cycling performance of the Se-R800A at 2 A g^−1^

Figure 5a shows the CV curves in the range of 0.005–3.0 V at a scan rate of 0.2 mV s^−1^. In the initial discharge process, in addition to the peak near 0 V which is the adsorption of Na^+^ at the defect sites and micropores of carbon matrix [44] like R800A and R800 samples, only a cathodic peak appears at about 1.2 V, indicating that the conversion of Se into Na_2_Se (Se ↔ Na_2_Se) is only a one-step reaction, which is very different from the mechanism of multistep reactions (Se ↔ Na_2_Se_*n*_, 3 < *n* < 8 ↔ Na_2_Se) between Se and Na [21, 24, 39]. And then the peak shifts to a more steady 1.1 V resulting from the electrochemical activation process [27]. For the charge process, only one anodic peak is observed and remains steady at 1.7 V in three cycles, indicating that it is a direct transformation of Na_2_Se into Se (Na_2_Se ↔ Se) at 1.7 V; therefore, the Se-R800A delivers to effectively facilitate suppression of the shuttle effect and maintenance of the specific capacity. The discharge/charge voltage profiles at 50 mA g^−1^ show the same trends in Fig. 5b, which coincides with the CV analysis. The single plateau is related to the conversion of Se to insoluble Na_2_Se [27]. The charge curves almost overlap during three cycles, while discharge curves alter from the initial with a capacity of 1100 mA h g^−1^ to the following cycles with a reversible capacity of 700 mA h g^−1^. It might account for the formation of the solid electrolyte interface (SEI) film on Se-R800A with partly irreversible trapping of Na^+^ in the pores [22]. Following the subsequent cycles, discharge curves overlap together as well, demonstrating that the Se-R800A achieves the superior cycling stability. The cycling performance of the Se-R800A electrode is shown in Fig. 5c. The capacity delivered initial capacity of 620 mA h g^−1^ and retained 520 mA h g^−1^ at 100 mA g^−1^ after 120 cycles, which is 84% of the theoretical capacity of Se, indicating the excellent cycling stability, and the coulombic efficiency was maintained 100% except for that the initial coulombic efficiency is under 80% due to Na^+^ trapped in the porous biochar. By contrast, the specific capacity of R800A is only 18 mA h g^−1^, which may be attributed to severe SEI resistance due to the tremendous specific surface area. It is worth mentioned that the specific capacity of R800 is 80 mA h g^−1^ at 100 mA g^−1^ after 120 cycles but exceptionally stable, validating the unique natural superiority of multilayer biomass-derived materials is critical to extend Na-Se batteries lifespan. The rate performance of the Se-R800A electrode at different current densities is further investigated and shown in Fig. 5d. As the current density increased from 20, 60, 100, 200, 300 to 600 mA g^−1^, the Se-R800A electrode provided a specific capacity was from 745, 674, 655, 610, 573 to 486 mA h g^−1^, respectively. When the current density was set to 20 mA g^−1^, the reversible capacity recovered to 711 mA h g^−1^, delivering a remarkable rate capability of the electrode. Importantly, even at a high current density of 2 A g^−1^, the Se-R800A still delivered an excellent high reversible capability of 300 mA h g^−1^ after 500 long cycles with no capacity fading (Fig. 5e). This superior specific capacity and rate performance surpass most reported typical C-Se cathodes for Na-Se batteries (Table 1).

**Table 1:** The comparison of cycling performance for the C-Se cathodes for Na-Se batteries reported in literature

Materials	Current density (A g^−1^)	Reversible capacity (mA h g^−1^)	References
Se@PCNFs	0.05	520 at 80th cycle	[51]
C/Se	0.1	258 at 50th cycle	[52]
Se/C	0.1695	340 at 380th cycle	[38]
Se/(CNT@MPC)	0.678	441 at 100th cycle	[53]
Se@MCNFs	0.5	430 at 300th cycle	[31]
Se@CNFs-CNT	0.5	410 at 240th cycle	[34]
CNF/Se	0.339	478 at 200th cycle	[27]
Se-MnMC-B	0.0678	535 at 150th cycle	[54]
CPAN/Se	0.203	410 at 300th cycle	[55]
Se-NCMC	0.1356	400 at 150th cycle	[24]
Se-R800A	*0.1*	*520 at 120th cycle*	*This work*

It is noteworthy that the cycling stability at high current density, even at 2 A g^−1^, is better than that at 0.1 A g^−1^. This may be due to the following reasons: (i) the inartificially hierarchical biochar and moderate graphitization degree of the Se-R800A tremendously accelerate the Na^+^ and e^−^ transport to activate amorphous Se, therefore ensuring facile electrochemical kinetics even at high current density; (ii) the intermediates (Na_2_Se_*n*_, 3 < *n* < 8) at low current density have more chances to dissolve into the carbonate electrolyte, but the polyselenides are firmly confined in the micropores and retained by overlapping carbon sheets, which is effective to alleviate the shuttle effect, resulting in a high efficient utilization of Se during the long-term cycling [27].

**Fig. 6:**
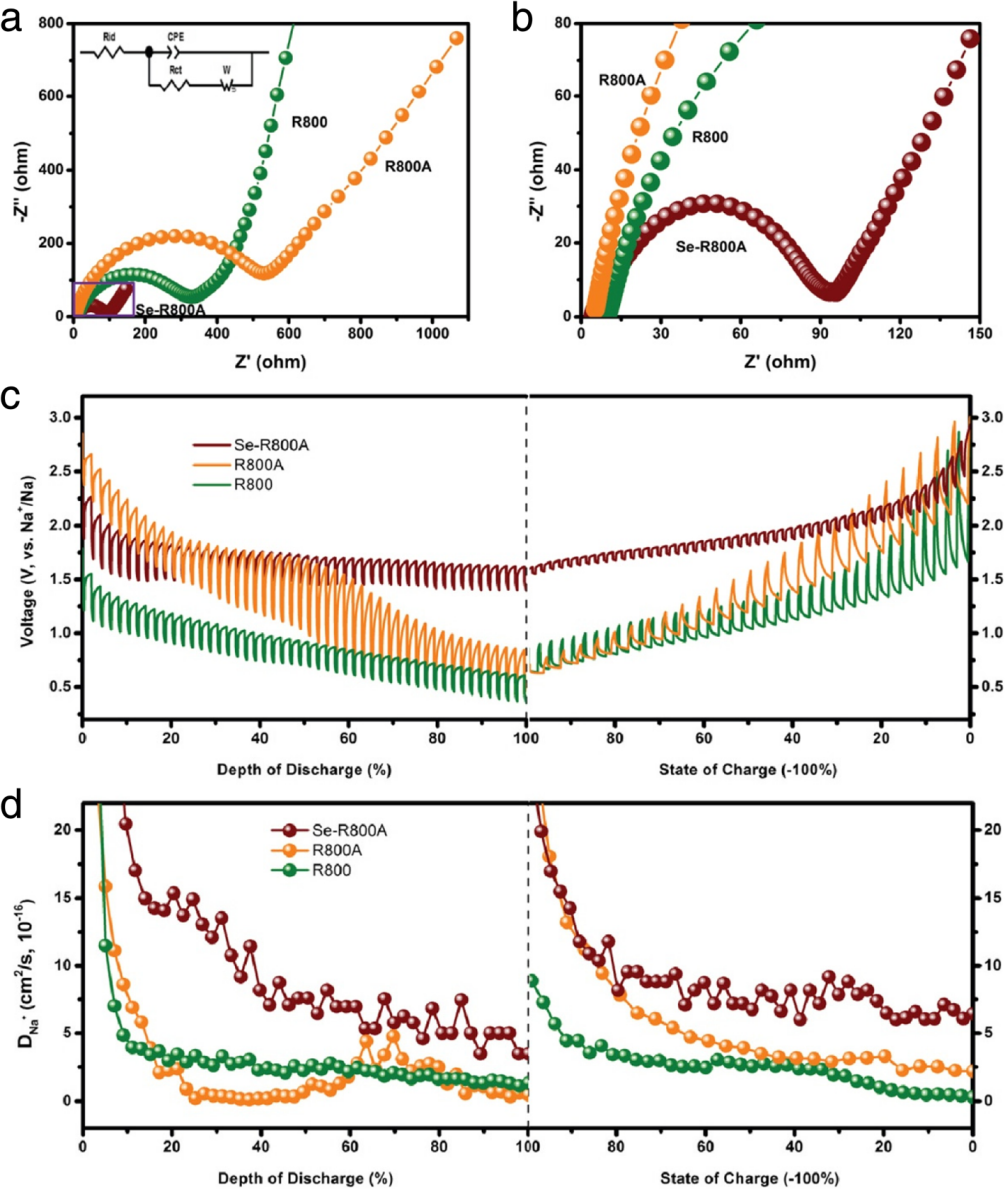
**a** Nyquist plots of Na-Se batteries assembled with the R800, R800A, and Se-R800A as cathodes for impedance analysis and the inset is the equivalent circuit. **b** Magnified the section of the Se-R800A. **c** Voltage profiles and **d** the Na^+^ diffusion coefficients of the R800, R800A, and Se-R800A obtained via the GITT technique during discharge/charge processes

In order to gain further information about the improved electrochemical performance of the Se-R800A, the charge-transfer resistance (*R*_ct_) and ion-diffusion resistance (*R*_id_) of the R800, R800A, and Se-R800A were measured by EIS. As shown in Fig. 6, the Nyquist plot of the R800 cathode exhibits the semicircle in the high-frequency regions attributed to *R*_ct_ containing the SEI layer and electrode-electrolyte interface [45, 46] and a sloping line in the low-frequency region corresponding to *R*_id_ representing the impedance of Na^+^ diffusion [47]. The R800A electrode presents larger radius semicircle after activation by KOH, indicating that abundant micropores contribute to accelerating the kinetic process of the electrochemical reactions but will distinctly increase the SEI layer resistance due to the tremendous surface area [22, 27, 31, 39, 40]. Furthermore, compared with the R800 electrode in Table 2, the smaller *R*_id_ implies micropores as ion-buffering reservoirs efficiently shorten ion-diffusion distance.

**Table 2:** The resistance values were obtained by modeling the equivalent circuit for experimental impedance

Materials	*R*_id_ (ohm)	*R*_ct_ (ohm)
R800	10.07	320.2
R800A	4.87	575.4
Se-R800A	4.33	89.44

When the loaded Se occupies the most of micropores, the Se-R800A electrode shows obviously smaller *R*_ct_ and *R*_id_ confirmed by the excellent electrochemical performance. The pores of the carbonized leaf are most in the range of 0.1–2 nm, and these abundant micropores are more suitable to load and confine Se, finally bringing a moderate surface area for higher coulombic efficiency [31, 37]. The Na^+^ diffusion coefficients of the three samples are calculated by the GITT method during discharge/charge process in Fig. 6c, d [48]. It can be observed that the Na^+^ diffusion coefficients of R800, R800A, and Se-R800A are the same order of magnitude (10^−16^ cm^2^/s) but the Se-R800A is higher than the others, which reveals that the Na^+^ diffusion in the carbon matrixes is notably improved due to the presence of Se [49, 50]. Together with these properties, both the electronic conductivity and the ionic diffusion efficiency in the carbon-selenium composite were effectively enhanced, resulting in an excellent electrochemical performance of Se-R800A electrode for Na-Se batteries.

**Fig. 7:**
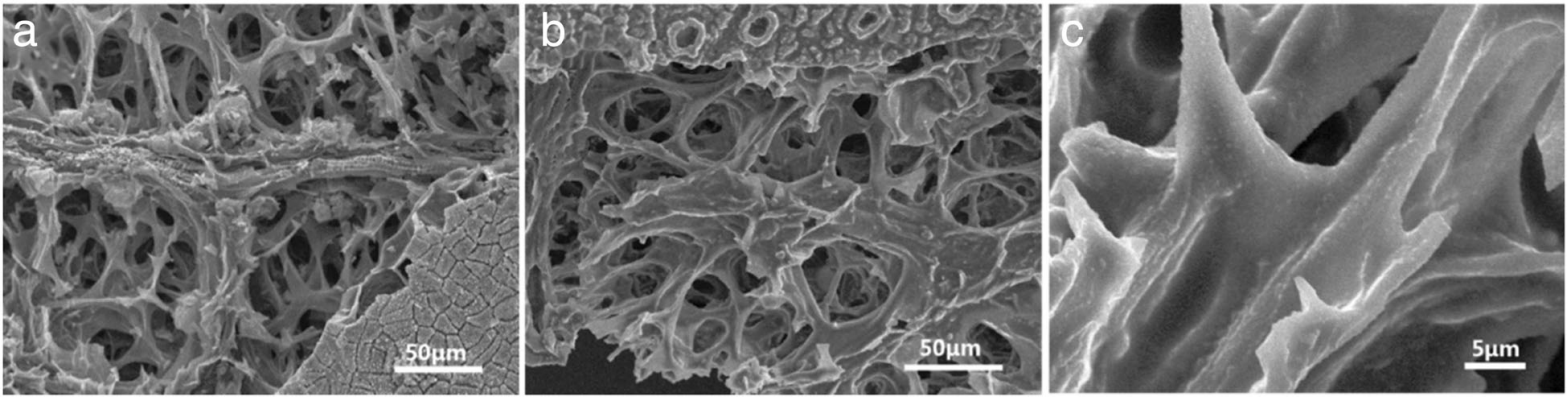
SEM images of the Se-R800A electrode film **a** before and **b** after cycles. **c** Magnified SEM image of the carbon sheet after cycles

After disassembling the testing cell, the morphology of the Se-R800A (Fig. 7b) keeps the same as the anteriority (Fig. 7a), suggesting that the carbonized leaf is qualified to serve as a favorable framework for Na-Se batteries. Figure 7c shows the hierarchical structure of the Se-R800A electrode after 500 cycles, and it maintained the original morphology as well. Therefore, it can be pointed out that the excellent cycling and rate performance can be due to the following reasons: firstly, the free-standing Se-R800A with abundant heteroatoms (such as N, O) for accommodating Se can suppress the shuttle effect of the polyselenides. Secondly, the hierarchical structure of the carbonized leaf with anisotropic surface could meet the need of the e^−^ and Na^+^ transport to activate inner Se. The polyselenides are confined in the micropores and retained by overlapping carbon sheets to increase the energy barrier of the polyselenide diffusion [42]. Finally, as binder- and current collector-free cathodes, the 3D interconnected framework and interlinked carbon sheets can profoundly facilitate the electrolyte infiltration and shorten ions diffusion distance [22]. These advantages are critical for the enhanced capacity and extended lifespan.

## Conclusions

In conclusion, it was demonstrated that a novel fabrication of the Se-R8000A can be finished by a tube furnace. It was successful to confine Se into the microporous carbonized leaf by common melt-infusion methods, which can effectively reduce the shuttle effect of polyselenides, resulting in excellent electrochemical performance for Na-Se batteries. The Se-R8000A shows a reversible capacity as high as 520 mA h g^−1^ at 100 mA g^−1^ after 120 cycles, which supports the superior cycling stability and rate capability. The inartificially hierarchical leaf structure and moderate graphitization degree of the Se-R800A were proved to significantly promote the efficient utilization of Se. Generally, the Se-R800A, owing to the free-standing, high-performance, and cost-effective characteristics, was demonstrated to be a promising alternative to conventional and substantial electrode materials in Na-Se batteries.

## Abbreviations

BET: Brunauer-Emmett-Teller
CV: Cyclic voltammogram
DEC: Diethyl carbonate
EC: Ethylene carbonate
EIS: Electrochemical impedance spectroscopy
FESEM: Field emission scanning electron microscopy
GITT: Galvanostatic intermittent titration technique
LIBs: Lithium ion batteries
Li-S: Lithium-sulfur
Na-Se: Sodium-selenium
SEI: Solid electrolyte interface
SEM: Scanning electron microscopy
SIBs: Sodium ion batteries
TEM: Transmission electron microscopy
TGA: Thermogravimetric analysis
XPS: X-ray photoelectron spectroscopy
XRD: X-ray diffraction
